# Amalgam for Tooth Filling

**Published:** 1879-01

**Authors:** 


### ARTICLE V.

### *Amalgam for Tooth Filling.*

A forceful argument in favor of the use of amalgam under many conditions appears in our excellent contemporary, the *Dental Cosmos* for December, from the pen of D. Van

Denburgh, of San Francisco. The writer was educated in the Parmly, Harris, and Westcott school of opinion that amalgam was unfit for tooth filling. His use of it began in little faith or hope that it would prove serviceable; and the fact that amalgam had won a place of usefulness in his practice is accounted evidence of its merits. He says: "Many of my own teeth were filled by C. A. Harris, of Baltimore, thirty years ago. A few more in the half dozen succeeding years were filled by Amos Westcott, of Syracuse, N. Y. One alone of these fillings failed, and that repeatedly within the first half dozen years. I would not have the tooth extracted, as advised; and, after having the decay excavated, I prepared a putty of tin, silver, and quicksilver, and myself placed it in the cavity of the unfortunate lower molar tooth. For more than twenty years it has done me as good service as its brighter neighbors. But the point of interest is, that it has done this where gold skillfully used had failed. After a year or two of good conduct by this amalgam filling, I began to feel some interest in it, and in various ways in my laboratory to test some amalgam mixtures.

"I occasionally filled in some mouths (where I might have opportunities afterwards to watch it) some dilapidated tooth that would otherwise have been extracted, some soft or poorly cared for teeth that fillings of any kind were least likely to preserve, and some difficult cavities that were hard to fill satisfactorily with gold. With such a beginning I went on experimenting, testing and comparing, and now, after twenty years, I still use it, and nearly as often as I use gold."

The mixture used by Mr. Van Denburgh is composed of coin silver, 4 parts; tin, 5 parts; and mercury, *q. s* The tin is added to prevent the shrinkage experienced with an amalgam of silver, and to form a mass sufficiently hard to resist mastication. The amalgamation is a matter requiring great care and skill, and thorough washing with alcohol is essential.

In the earlier years of his use of amalgam, Mr. Van Denburgh thought he occasionally observed so much superficial oxidation, as, after a considerable time, to injure the edges of certain fillings, and produce a crevice that favored the renewal of decay; but longer experience has taught him that the fault was his own in unskillful preparation and use of the material. He believes now that amalgam will resist all destructive agents in the mouth as well as any dentine or enamel can do; but a caution may be necessary to the inexperienced, not to use so much or so little quicksilver as to make it crumble under mastication; or to attempt to use the mixture in any case after it may be perceived that the hardening process has begun. If this process be once interfered with, though it will be renewed in a degree, no reliance can be placed upon the mixture. A good amalgam filling will not stain any tooth. That anything hurtful to the most delicate or diseased organization can be found in it, or produced from it in the mouth, seems to him the most fanciful nonsense. Any quantity of the metal that might be placed in the mouth would not probably lose by trituration or chemical action a perceptible amount of any or all of its ingredients in a lifetime; and even if such loss were to occur in any way or form, he knows of no reason to suppose that it would be hurtful if taken into the stomach.

As regards durability, Mr. Van Denburgh believes that properly prepared amalgam, skillfully inserted, provides as lasting a filling as can be made with gold, and its advantages in saving time and pain are very great. He says:

"Five minutes are sufficient to insert any amalgam filling, and nothing more is required to keep the work dry than a napkin or a roll of paper. But not only am I convinced that amalgam, in respect to usefulness and durability, may in any case be as good as gold; I am equally certain that in some cases it is superior, and for the reason I have mentioned, of its more kindly contact and healing qualities in connection with soft dentine. As a mechanical stopping, where a tooth is much weakened by the loss of substance, amalgam brings no strain as gold does; on the contrary, it becomes a binding and supporting protection. Much of the solid substance of teeth is often cut away to sucure the better insertion of gold that would be unnecessary for amalgam. Any one with strong masticating organs can 'chew up' a gold coin; so, the most perfect gold filling, when made a projecting portion of a tooth, and subjected to severe mastication, will spread and finally break away. Amalgam has a greater power of resistance, and in many such cases will prove the most serviceable grinder."

It is but fair to note that in the same number of the *Dental Cosmos*, Dr. William H. Truman, of Philadelphia, critically reviews the question of amalgam fillings, and arrives at conclusions much less favorable to their use, though he is far from condemning their use under all conditions. The materials proposed in place of gold will no doubt amply repay by increased durability any extra care in their preparation and insertion; but they all, he thinks, have inherent defects which will have to be overcome before they can be called reliable. They have their place, and probably have not been used as much as they might. Nevertheless, "gold has been used too long, and with too much success, to be abandoned before a better method is at hand. We would ask every professional brother before accepting the teaching and adopting the practice of this new departure, to reflect a moment on the past, and see how often these tidal waves have swept over the profession. At one time it is arsenic, at another amalgam; twice sponge gold has rolled over us, leaving many an aching void; and no doubt the indiscriminate use of the mallet and cohesive gold, the reckless building up o teeth without regard to their position or character, has done much to secure this idea the warm reception it has met."

Accordingly, Dr. Truman advises caution before cutting loose from so faithful and well-tried a friend and risking professional reputation on "the uncertain ground of plastic fillings." The fault *may* lie with the operator, and not with the material used.—*Scientific American.*

